# SH3BP2 Gain-Of-Function Mutation Exacerbates Inflammation and Bone Loss in a Murine Collagen-Induced Arthritis Model

**DOI:** 10.1371/journal.pone.0105518

**Published:** 2014-08-21

**Authors:** Tomoyuki Mukai, Richard Gallant, Shu Ishida, Teruhito Yoshitaka, Mizuho Kittaka, Keiichiro Nishida, David A. Fox, Yoshitaka Morita, Yasuyoshi Ueki

**Affiliations:** 1 Department of Oral and Craniofacial Sciences, School of Dentistry, University of Missouri-Kansas City, Kansas City, Missouri, United States of America; 2 Department of Periodontal Medicine, Applied life Sciences, Institute of Biomedical and Health Sciences, Graduate School of Biomedical and Health Sciences, Hiroshima University, Hiroshima, Japan; 3 Department of Orthopaedic Surgery, Okayama University Graduate School of Medicine, Dentistry and Pharmaceutical Sciences, Okayama, Japan; 4 Department of Human Morphology, Okayama University Graduate School of Medicine, Dentistry and Pharmaceutical Sciences, Okayama, Japan; 5 Division of Rheumatology, Department of Internal Medicine, University of Michigan, Ann Arbor, Michigan, United States of America; 6 Department of Rheumatology, Kawasaki Medical School, Kurashiki, Japan; University of Leicester, United Kingdom

## Abstract

**Objective:**

SH3BP2 is a signaling adapter protein which regulates immune and skeletal systems. Gain-of-function mutations in SH3BP2 cause cherubism, characterized by jawbone destruction. This study was aimed to examine the role of SH3BP2 in inflammatory bone loss using a collagen-induced arthritis (CIA) model.

**Methods:**

CIA was induced in wild-type (*Sh3bp2^+/+^*) and heterozygous P416R SH3BP2 cherubism mutant knock-in (*Sh3bp2^KI/+^*) mice, an SH3BP2 gain-of-function model. Severity of the arthritis was determined by assessing the paw swelling and histological analyses of the joints. Micro-CT analysis was used to determine the levels of bone loss. Inflammation and osteoclastogenesis in the joints were evaluated by quantitating the gene expression of inflammatory cytokines and osteoclast markers. Furthermore, involvement of the T- and B-cell responses was determined by draining lymph node cell culture and measurement of the serum anti-mouse type II collagen antibody levels, respectively. Finally, roles of the SH3BP2 mutation in macrophage activation and osteoclastogenesis were determined by evaluating the TNF-α production levels and osteoclast formation in bone marrow-derived M-CSF-dependent macrophage (BMM) cultures.

**Results:**

*Sh3bp2^KI/+^* mice exhibited more severe inflammation and bone loss, accompanying an increased number of osteoclasts. The mRNA levels for TNF-α and osteoclast marker genes were higher in the joints of *Sh3bp2^KI/+^* mice. Lymph node cell culture showed that lymphocyte proliferation and IFN-γ and IL-17 production were comparable between *Sh3bp2^+/+^* and *Sh3bp2^KI/+^* cells. Serum anti-type II collagen antibody levels were comparable between *Sh3bp2^+/+^* and *Sh3bp2^KI/+^* mice. In vitro experiments showed that TNF-α production in *Sh3bp2^KI/+^* BMMs is elevated compared with *Sh3bp2^+/+^* BMMs and that RANKL-induced osteoclastogenesis is enhanced in *Sh3bp2^KI/+^* BMMs associated with increased NFATc1 nuclear localization.

**Conclusion:**

Gain-of-function of SH3BP2 augments inflammation and bone loss in the CIA model through increased macrophage activation and osteoclast formation. Therefore, modulation of the SH3BP2 expression may have therapeutic potential for the treatment of rheumatoid arthritis.

## Introduction

Rheumatoid arthritis (RA) is a chronic systemic inflammatory disease resulting in bone loss. Bone loss is exclusively driven by osteoclasts [1], [2], which are bone-resorbing cells derived from myeloid cells. Osteoclast differentiation is regulated mainly by receptor activator of nuclear factor-κB (RANK) and its ligand, RANKL. RANKL is predominantly expressed on osteoblasts and osteocytes [3]–[5]. In pathological conditions, it can be expressed by other cells such as fibroblasts and T cells [6]–[8]. In RA, inflammatory cytokines including tumor necrosis factor (TNF)-α, interleukin (IL)-1β, and IL-6, have been found to enhance RANKL expression in synovial fibroblasts, which subsequently activates osteoclasts in joints [6]–[8]. Additionally, these inflammatory cytokines can enhance osteoclastogenesis in cooperation with RANKL [9], [10]. As a result, excessive osteoclast activity causes bone loss in inflamed joints and throughout the body, leading to the loss of joint motion and increased risk of fractures in RA patients [11]. However, further research is needed to fully elucidate the pathophysiology of osteoclast-driven bone loss in RA.

SH3 domain-binding protein 2 (SH3BP2) is an adaptor protein, which is expressed primarily in immune cells including T cells, B cells, mast cells, neutrophils, and macrophages as well as osteoclasts [12]–[17]. SH3BP2 interacts with various proteins, including SYK [18], PLCγ [18], [19], SRC [20], [21], and VAV [22], and regulates intracellular signaling pathways in immune and skeletal systems [13]–[17], [23]. Previously we have reported that gain-of-function mutations in SH3BP2 are responsible for cherubism (OMIM#118400) [24]. Cherubism is an autosomal dominant craniofacial disorder characterized by excessive jawbone destruction with swelling of the lower face [25]. The jaw lesions consist mostly of fibroblastoid cells with a large number of tartrate-resistant acid phosphatase (TRAP)-positive multinucleated giant cells [25], [26], suggesting that the excessive jawbone resorption is caused by increased osteoclastogenesis.

We have introduced a P416R SH3BP2 mutation (equivalent to the most common P418R mutation in cherubism patients) into the mouse genome and established a knock-in (KI) mouse model for cherubism [23]. Analysis of the mouse model has revealed that homozygous mutant (*Sh3bp2^KI/KI^*) mice spontaneously develop severe arthritis and osteopenia and that heterozygous (*Sh3bp2^KI/+^*) mice exhibit mild osteopenia with no obvious inflammation [23]. More recently it has been reported that SH3BP2 interacts with poly(ADP-ribose) polymerase family members, Tankyrase 1 and Tankyrase 2, and that cherubism mutations disrupt the binding of SH3BP2 to the Tankyrases, resulting in inhibition of Tankyrase-mediated SH3BP2 protein degradation [12], [27]. Therefore, SH3BP2 cherubism mutations increase SH3BP2 protein levels in mutant cells and cause cherubism in a gain-of-function manner. This concept is supported by our previous finding that overexpression of either wild-type or P416R SH3BP2 protein in primary wild-type bone marrow-derived M-CSF-dependent macrophages (BMMs) enhances RANKL-induced osteoclastogenesis, which is similar to RANKL-stimulated *Sh3bp2^KI/+^* and *Sh3bp2^KI/KI^* BMMs [23].

The finding that the homozygous *Sh3bp2^KI/KI^* mice exhibit severe inflammatory joint destruction and osteopenia prompted us to consider a possible involvement of SH3BP2 in the bone erosion process in RA. Although the heterozygous *Sh3bp2^KI/+^* mice do not develop arthritis [23], expression of SH3BP2 protein is elevated in *Sh3bp2^KI/+^* BMMs [12]. Therefore, we hypothesized that the *Sh3bp2^KI/+^* mice are susceptible to the stimulus that causes arthritis and bone destruction. In the present study, we investigated a role of SH3BP2 in inflammatory bone loss in an arthritis model that requires adaptive immune responses. We introduced collagen-induced arthritis (CIA) in the non-arthritic heterozygous *Sh3bp2^KI/+^* mice and examined whether SH3BP2 gain-of-function affects the development of CIA. We utilized *Sh3bp2^KI/+^* mice as an SH3BP2 gain-of-function model, because reduction in survival rate in *Sh3bp2^KI/KI^* mice due to severe systemic organ inflammation [23] hinders the long-term observation of arthritis. Here we demonstrate that the SH3BP2 gain-of-function mutation augments inflammation and focal bone loss in joints as well as systemic bone loss via increased activation of macrophages and osteoclast formation in the CIA model.

## Materials and Methods

### Ethics statement

This study was performed in strict accordance with the recommendations in the Guide for the Care and Use of Laboratory Animals of the National Institutes of Health. The protocol was approved by the Institutional Animal Care and Use Committees of the University of Missouri-Kansas City (UMKC) (permit number: 1144e).

### Mice

SH3BP2 P416R cherubism knock-in mutant mice were generated by introducing a proline-to-arginine mutation into exon 9 of the murine *Sh3bp2* gene as reported previously [23]. Heterozygous and homozygous mice carrying the P416R cherubism mutation in *Sh3bp2* gene are referred to as *Sh3bp2^KI/+^* and *Sh3bp2^KI/KI^* mice, respectively. DBA/1 mice were purchased from the Jackson Laboratory (Bar Harbor, ME, USA). All mice in this study were housed in a specific pathogen-free facility at UMKC.

### Reagents

Chick type II collagen (CII), complete Freund’s adjuvant (M. tuberculosis 3****mg/ml), incomplete Freund’s adjuvant, denatured chick CII, and anti-mouse CII antibody assay kits for total IgG, IgG1, IgG2a, and IgG2b were purchased from Chondrex (Redmond, WA, USA). Recombinant murine macrophage colony-stimulating factor (M-CSF) and RANKL were obtained from Peprotech (Rocky Hill, NJ, USA). Anti-SH3BP2 antibody (clone: 1E9, Abnova, Taipei City, Taiwan), anti-NFATc1 antibody (clone: 7A6, Santa Cruz Biotechnology, Dallas, TX, USA), anti-actin antibody (Sigma-Aldrich, St. Louis, MO, USA), anti-nuclear matrix protein p84 antibody (clone: 5E10, abcam, Cambridge, MA, USA), and anti-heat shock protein 90 (HSP90) antibody (Cell Signaling Technology, Danvers, MA, USA) were used.

### Study design for the CIA experiment

Before the induction of CIA, SH3BP2 P416R mutant mice (C57BL/6 background) were backcrossed for 10 generations onto DBA/1 genetic background to create a congenic strain with P416R SH3BP2 mutation on the DBA/1 background. Since DBA/1 background is more susceptible to CIA than C57BL/6 background [28], [29], this strain allows us to induce the arthritis effectively, resulting in more reliable CIA study with less variation in data.

**Figure 1:**
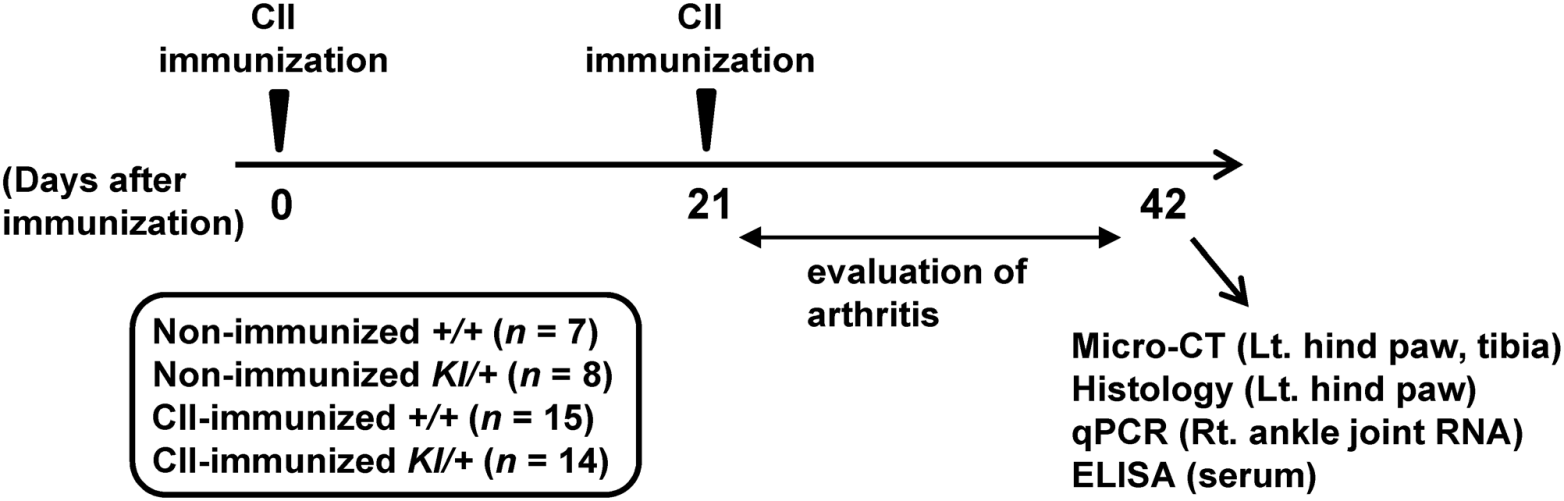
Study design of the CIA experiment. Nine-week-old male SH3BP2 wild-type (*Sh3bp2^+/+^*) (*n* = 15) and P416R SH3BP2 knock-in heterozygous (*Sh3bp2^KI/+^*) mice (*n* = 14) were immunized intradermally with chick type II collagen (CII) on day 0, and CII boost injections were given on day 21. Swelling of the paws was evaluated until day 42 as described in the Materials and Methods. The mice were euthanized at day 42, and bone, RNA, and serum samples were collected. The left hind paws and tibiae were applied to the micro-CT analysis. The left hind paws were subsequently applied to the histological analysis. The right ankle joint tissues were used for RNA extraction for qPCR analysis. The serum samples were applied to the ELISA assay.

The experimental timeline for CIA is shown in Figure 1. CIA was induced in *Sh3bp2^+/+^* (*n* = 15) and *Sh3bp2^KI/+^* (*n* = 14) mice. Age- and sex-matched *Sh3bp2^+/+^* (*n* = 7) and *Sh3bp2^KI/+^* (*n* = 8) mice were used as non-immunized control *Sh3bp2^+/+^* and *Sh3bp2^KI/+^* mice. Nine-week-old male *Sh3bp2^+/+^* and *Sh3bp2^KI/+^* mice on the DBA/1 background were injected intradermally with 100 µg of chick CII in complete Freund’s adjuvant at the base of the tail on day 0 [29], [30]. On day 21, booster injection containing 100 µg of chick CII in incomplete Freund’s adjuvant was given. Arthritis severity was assessed twice each week in a blinded manner until day 42 using the criteria as follows [31]: 0 = normal; 1 = mild erythema or swelling of the wrist or ankle or erythema and swelling of any severity for 1 digit; 2 = moderate erythema and swelling of the ankle or wrist or more than three inflamed digits; 3 = severe erythema and swelling of wrist or ankle; 4 = complete erythema and swelling of the wrist and ankle including all digits. Each limb was graded, giving a maximum score of 16. Mice which exhibited 2 or higher arthritis score were included as arthritic mice. On day 42, mice were euthanized in a carbon dioxide (CO_2_) container. Immediately after euthanasia, blood samples were collected by cardiac puncture. The yielded serum samples were applied to ELISA assay. Right ankle joints were crushed in liquid nitrogen, stored at −80°C, and subsequently applied to RNA extraction and qPCR analysis. Left hind paws were harvested and applied to micro-computed tomographic (micro-CT) and histological analyses. Left tibiae were harvested and applied to micro-CT analysis. All CII-immunized and non-immunized *Sh3bp2^+/+^* and *Sh3bp2^KI/+^* mice were included in the ELISA, qPCR, and micro-CT analyses; arthritic *Sh3bp2^+/+^* (*n* = 11 out of 15) and *Sh3bp2^KI/+^* (*n*  = 10 out of 14) mice were included in the quantitative histological analyses. Detailed procedures for individual experiments are described in the following sections.

### Micro-CT analysis

Left hind paws and tibiae were fixed in 4% paraformaldehyde (PFA) in PBS (pH 7.4) for 2 days and then stored in 70% ethanol until micro-CT analysis. The PFA-fixed hind paw and tibia were separately placed in a microcentrifuge tube with 70% ethanol and scanned with viva CT40 (Scanco Medical AG, Bassersdorf, Switzerland) with an X-ray energy of 55 kVp (145 µA), a voxel resolution of 15 µm, and an integration time of 200 ms. The threshold was set to 300 (equivalent to 471.1 mg hydroxyapatite (HA)/cm^3^) for the hind paw, 260 (384.7 mg HA/cm^3^) for the cortical bone of the tibia, and 220 (298.2 mg HA/cm^3^) for the trabecular bone of the tibia to distinguish mineralized tissues from non-mineralized tissues. The talar bone volume was evaluated for a quantitative measurement of bone erosion [32]. The region of cortical bone comprised 30 slices of midshaft (1 mm proximal to the tibio-fibular junction); the region of trabecular bone comprised 70 slices of secondary spongiosa beginning just beneath primary spongiosa. Bone properties were analyzed using Scanco bone evaluation software. All micro-CT analysis procedures were performed according to the international guideline [33]. After the analysis, bone samples were stored in 70% ethanol at 4°C until histological analysis.

### Histological analysis

For histological analysis, the hind paw samples were decalcified in 0.5 M EDTA (pH 7.2) for 4 weeks at 4°C and embedded in paraffin. Sections (6****µm) were stained with hematoxylin and eosin (H&E) and Safranin O (pH 7.4). Severity of the inflammation and cartilage damage around talus bones was evaluated in a blinded manner by two independent observers using scoring systems. The severity of inflammation was determined by the area of inflammatory cells infiltrates: 0 = no significant inflammatory cell infiltrates, 1 = mild diffuse inflammatory cell infiltrates, 2 = moderate inflammatory cell infiltrates, 3 = marked inflammatory cell infiltrates, 4 = severe inflammatory cell infiltrates with pannus formation. The severity of cartilage damage was determined by the loss of Safranin O-stained area in the cartilage, which indicates the presence of glycosaminoglycan, and chondrocyte loss: 0 = no significant loss of Safranin O staining area, 1 = mild loss of Safranin O staining with no obvious chondrocyte loss, 2 = moderate loss of Safranin O staining with focal mild (superficial) chondrocyte loss, 3 = marked loss of Safranin O staining with multifocal marked chondrocyte loss, 4 = severe diffuse loss of Safranin O staining with multifocal severe chondrocyte loss.

### Histomorphometric analysis

TRAP staining counterstained with methyl green was performed to visualize TRAP-positive cells. Histomorphometric measurements were performed in a blinded manner using OsteoMeasure computerized image analysis system (OsteoMetrics Inc., Atlanta, GA, USA) [34]. TRAP-positive multinucleated cells (3 or more nuclei) were defined as osteoclasts. Bone erosion on the surface of the talus was traced and attached osteoclasts were counted. Eroded surface per bone surface (ES/BS) and number of osteoclasts per bone surface (N.Oc/BS) of the talus were determined. The terminology and units were described according to international guidelines [35].

### Real-time quantitative RT-PCR

RNA samples were extracted from whole joints as previously reported [36] with some modifications. Immediately after euthanasia and blood collection, right ankle tissues were harvested by cutting the distal end of tibia and metatarsal bones. After removing skin, whole joint tissues including synovium and bones were crushed under liquid nitrogen using a tissue pulverizer (The Cellcrusher, Cellcrusher Limited, Portland, OR, USA). The resulting powder was collected in microcentrifuge tubes containing TRIzol (Life Technologies, Carlsbad, CA, USA) and stored at −80°C until use. Total RNA was extracted according to the manufacturer’s protocol. The extracted RNA (500****ng/sample) was transcribed to cDNA using High Capacity cDNA Reverse Transcription Kits (Life Technologies). qPCR reactions were performed with StepOne Plus system (Life Technologies) using Absolute Blue QPCR Master Mixes (Thermo Scientific, Waltham, MA, USA). Gene expression levels relative to *Hprt* were calculated by the ΔΔCt method as described [34], [37]. Average gene expression levels in joint tissues from non-immunized wild-type mice and wild-type BMMs at 0 hour were set as 1 to normalize the expression levels (fold-change). Primers used in this study are listed in Table 1. All primers were purchased from Integrated DNA Technologies, Inc. (Coralville, IA, USA). All qPCR reactions yielded products with single peak dissociation curves.

**Table 1:** qPCR primers used in this study.

Primer	Gene accessionnumber	Sequence	Ampliconsize (bp)	Annealingtemperature (°C)
*Tnfa*	NM_013693.2	Forward: 5′-catcttctcaaaattcgagtgaca-3′Reverse: 5′-tgggagtagacaaggtacaaccc-3′	175	60
*Il1b*	NM_008361.3	Forward: 5′-agttgacggaccccaaaag-3′Reverse: 5′-agctggatgctctcatcagg-3′	75	60
*Il6*	NM_031168.1	Forward: 5′-aacgatgatgcacttgcaga-3′Reverse: 5′-ccagaggaaattttcaataggc-3′	113	60
*Acp5*	NM_001102405.1	Forward: 5′-cagcagcccaaaatgcct-3′Reverse: 5′-ttttgagccaggacagctga-3′	61	60
*Ctsk*	NM_007802.3	Forward: 5′-cgaaaagagcctagcgaaca-3′Reverse: 5′-tgggtagcagcagaaacttg-3′	67	60
*Oscar*	NM_175632.2	Forward: 5′-tctgccccctatgtgctatca-3′Reverse: 5′-aggagccagaaccttcgaaac-3′	67	60
*Rankl*	NM_011613.3	Forward: 5′-tgaagacacactacctgactcctg-3′Reverse: 5′-ccacaatgtgttgcagttcc-3′	83	60
*Opg*	NM_008764.3	Forward: 5′-tgtccagatgggttcttctca-3′Reverse: 5′-cgttgtcatgtgttgcatttcc-3′	118	60
*Hprt*	NM_013556.2	Forward: 5′-tcctcctcagaccgctttt-3′Reverse: 5′-cctggttcatcatcgctaatc-3′	90	60

### Cell proliferation and cytokine production in draining lymph node cell culture

Lymph node cell culture was performed as described [38] with some modifications. Nine-week-old male mice were immunized with 100 µg of chick CII in complete Freund’s adjuvant as described above. At 10 days after the immunization, inguinal lymph nodes were isolated. As non-immunized controls, inguinal lymph nodes were isolated from age-matched *Sh3bp2^+/+^* and *Sh3bp2^KI/+^* male mice. Lymph node cells were cultured at a density of 4.0×10^5^ cells/well in 96-well U-bottom plates in RPMI1640 supplemented with 10% heat-inactivated FBS, 50 µM 2-mercaptoethanol, and 1% L-glutamine at 37°C under 5% CO_2_. The cells were stimulated with 50 µg/ml of denatured chick CII for 72 hours. Cell proliferation was measured using CellTiter96 Aqueous One Solution (MTS) reagent (Promega, Madison, WI, USA). After 3-hour incubation, absorbance of the media was measured at 490 nm, and background absorbance (from wells with no cells) was subtracted. IFN-γ and IL-17 levels in culture supernatant were determined by ELISA (R&D systems, Minneapolis, MN, USA).

### ELISA assay for anti-mouse CII antibody

CII-immunized *Sh3bp2^+/+^* and *Sh3bp2^KI/+^* male mice were euthanized in a CO_2_ container at day 42 after the initial immunization. Immediately after euthanasia, blood samples were obtained by cardiac puncture with a 25 G needle. The blood samples were collected in blood collection tubes containing serum clot activator (BD, Franklin Lakes, NJ, USA), and serum was isolated by centrifugation for 20 minutes at 3000*g* at 4°C. The serum samples were stored at −80°C in microcentrifuge tubes until used. Serum levels of anti-mouse CII antibody (total IgG, IgG1, IgG2a, and IgG2b) were measured according to the manufacturer’s protocol (Chondrex). Diluted serum samples were incubated in CII-coated 96-well plates at 4°C overnight. Bound IgG was detected by incubation with HRP-conjugated sheep anti-mouse IgG, followed by colorimetric detection with OPD substrate. Absorbance was measured at 490 nm. Antibody concentrations were calculated by referencing to the standard curve.

### In vitro mouse primary macrophage/osteoclast culture

Isolation and culture of primary bone marrow cells were performed as described [23], [34] with some modifications. Bone marrow cells were isolated from long bones of 10-week-old female mice and cultured on Petri dishes for 2–4 hours at 37°C under 5% CO_2_. Non-adherent bone marrow cells were re-seeded on 48-well plates at a density of 1.0×10^5^ cells/well and incubated for 2 days at 37°C under 5% CO_2_ in α-MEM/10% FBS containing M-CSF (25 ng/ml). After 2-day preculture, BMMs were re-stimulated with M-CSF (25 ng/ml). TNF-α production in culture supernatant was determined by ELISA (R&D systems). RNA samples from the BMMs were isolated using TRIzol and subjected to qPCR analysis for TNF-α and IL-1β mRNA expressions. For the osteoclast differentiation assay, BMMs were stimulated with RANKL at indicated concentrations for 3 days in the presence of M-CSF (25 ng/ml). TRAP-positive multinucleated cells (3 or more nuclei) were visualized by TRAP staining (Sigma-Aldrich) and counted at 40X magnification (*n* = 4–6 wells/group).

### Immunofluorescent staining

Immunofluorescent staining was performed as described previously [34]. Non-adherent bone marrow cells were seeded on 8-well chamber slides at a density of 1.8×10^5 ^cells/well. After 2-day preculture with M-CSF (25 ng/ml), BMMs were stimulated with RANKL (25 ng/ml) in the presence of M-CSF (25 ng/ml). The cells were fixed in 4% PFA, permeabilized with 0.2% Triton X-100, blocked in 2% normal goat serum/2.5% BSA/PBS, and incubated with anti-NFATc1 antibody. NFATc1 was detected by Alexa Fluor-555 conjugated goat anti-mouse IgG antibody (Life Technologies). Actin and nuclei were co-stained with Alexa Fluor-488 conjugated phalloidin (Life Technologies) and DAPI (Santa Cruz Biotechnology), respectively. Fluorescent images of the cells were acquired with Nikon E800 microscope (Nikon corp., Tokyo, Japan). To quantify nuclear localization of NFATc1, three images per each group were taken at 40X magnification. Cells were considered positive for NFATc1 nuclear localization when the fluorescence intensity of NFATc1 in nuclei exceeded that in cytoplasm. Numbers of DAPI- and NFATc1-positive nuclei were counted using ImageJ (NIH), and the percentages of NFATc1-positive nuclei per total nuclei were calculated as reported [39].

### Western blot

For protein sample preparation, BMMs were washed with ice-cold PBS and lysed with lysis buffer (25 mM Tris-HCl (pH 7.4), 150 mM NaCl, 5 mM EDTA, 10% glycerol, 1% Triton X-100, 2.5 mM sodium pyrophosphate, 0.7 mM β-glycerophosphate) with protease inhibitor and phosphatase inhibitor cocktails (Sigma-Aldrich). For the preparation of protein in tissues (lymph node, thymus, spleen, and heart), the tissues were harvested from 9-week-old *Sh3bp2^+/+^* and *Sh3bp2^KI/+^* male mice immediately after euthanasia. The tissues were minced, lysed with the lysis buffer described above, and sonicated for 20 sec on ice using a sonicator (Branson Ultrasonics, Danbury, CT, USA). After centrifugation (17000×*g* for 15 min, 4°C), supernatants were collected. Nuclear and cytoplasmic fractions were extracted as reported previously [34]. Four µg of TritonX-100-solubilized protein from BMMs and tissues and 1 µg of nuclear protein were resolved by SDS-PAGE and transferred to nitrocellulose membranes. After blocking with 5% skim milk in TBST buffer, membranes were incubated with primary antibodies followed by incubation with appropriate HRP-conjugated species-specific secondary antibodies (Cell Signaling Technology). Bands were detected using SuperSignal West Dura or Femto chemiluminescent substrate (Thermo Scientific) and visualized by ImageQuant LAS-4000 (GE Healthcare). Actin, HSP90, and nuclear matrix protein p84 were used as loading controls.

### Statistical analysis

All values are given as the mean ± SEM. Statistical analysis was performed by the two-tailed unpaired Student’s *t*-test to compare two groups or by one-way ANOVA (Tukey post-hoc test) to compare three or more groups. Incidence of arthritis was analyzed by Fisher’s exact test. GraphPad Prism 5 (GraphPad Software, San Diego, CA, USA) and SPSS Statistics 20 (IBM, Armonk, NY, USA) were used for all statistical analyses. *P* values less than 0.05 were considered statistically significant.

## Results

### Increased SH3BP2 protein expression in P416R SH3BP2 mutant BMMs and lymphoid tissues

**Figure 2:**
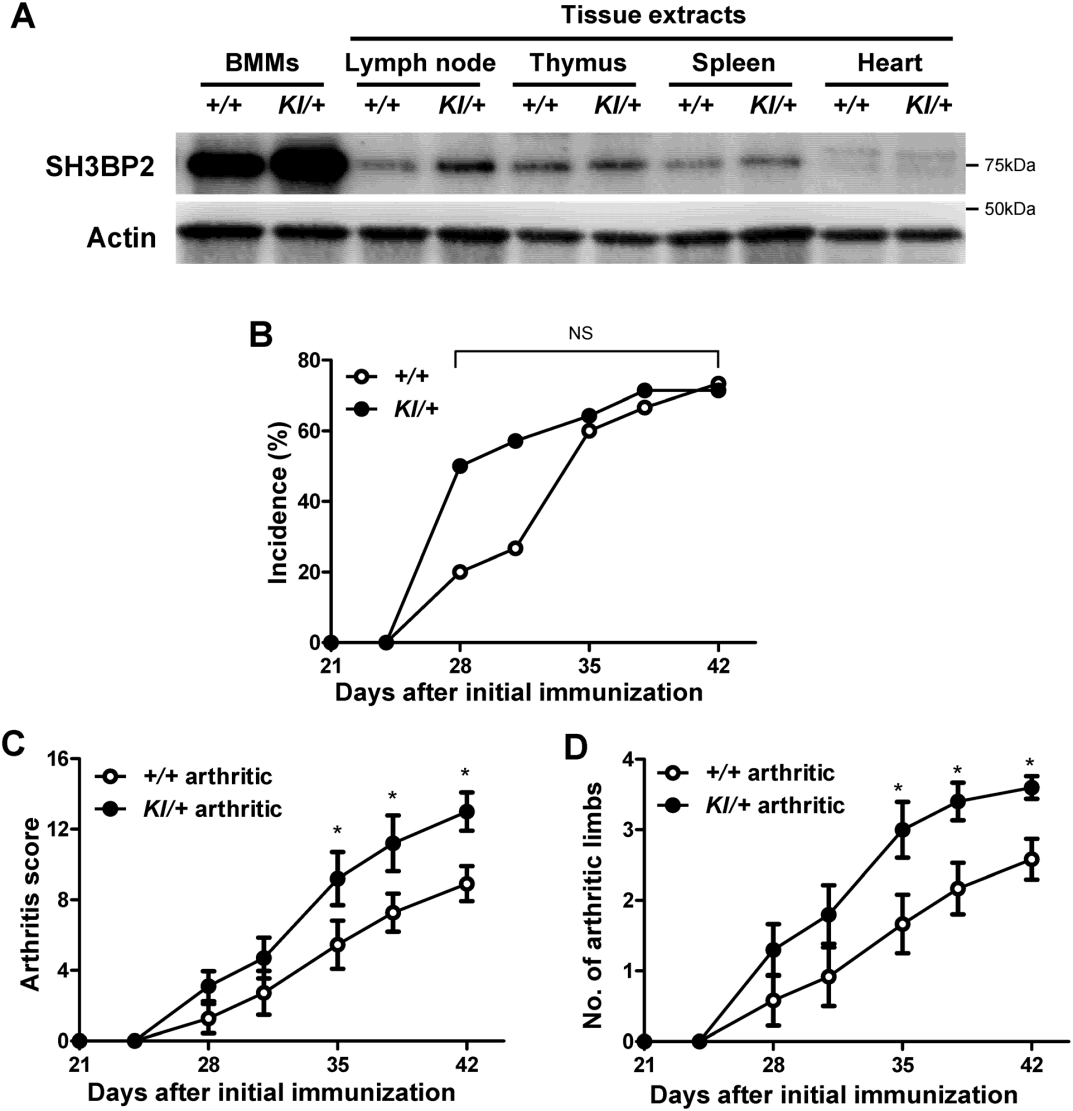
Increased severity of arthritis in P416R SH3BP2 knock-in mice. **A**, Western blot analysis for SH3BP2. Protein samples were extracted from *Sh3bp2^+/+^* and *Sh3bp2^KI/+^* bone marrow-derived M-CSF-dependent macrophages (BMMs) after 3-day culture with M-CSF (25****ng/ml) and from the indicated tissues from 9-week-old *Sh3bp2^+/+^* and *Sh3bp2^KI/+^* male mice. Four µg of protein samples were applied to each lane. Actin was used as a loading control. **B–D**, CIA was induced in 9-week-old male *Sh3bp2^+/+^* and *Sh3bp2^KI/+^* mice. Swelling of the paws was evaluated until day 42 as described in the Materials and Methods. Mice which exhibited 2 or higher arthritis score were included as arthritic mice. **B**, Cumulative incidence of arthritis in CII-immunized *Sh3bp2^+/+^* (*n* = 15) and *Sh3bp2^KI/+^* mice (*n* = 14). **C**, Arthritis score of arthritic *Sh3bp2^+/+^* (*n* = 11) and *Sh3bp2^KI/+^* mice (*n* = 10). **D**, Number of arthritic limbs of the arthritic mice. Values are presented as the mean ± SEM. *+/+* = *Sh3bp2^+/+^*; *KI/+* = *Sh3bp2^KI/+^*. * = *p*<0.05 versus *Sh3bp2^+/+^* mice; NS = not significant.

In BMMs, P416R SH3BP2 mutation functions in a gain-of-function manner due to increased SH3BP2 protein levels [12]. We first examined whether SH3BP2 protein is highly expressed in lymphoid tissues, which are sites of the pathological immune reaction in CIA development [40], [41]. Western blot analysis showed that SH3BP2 protein expressions are high in BMMs as well as in lymph node, thymus, and spleen tissues compared with that in heart in both *Sh3bp2^KI/+^* and *Sh3bp2^+/+^* mice (Figure 2A). Expressions were increased in *Sh3bp2^KI/+^* tissues compared with that in *Sh3bp2^+/+^* tissues (Figure 2A). These results suggest that P416R SH3BP2 mutation results in the elevated SH3BP2 protein levels not only in myeloid cells but also in lymphoid cells.

### Increased severity of arthritis in P416R SH3BP2 mutant mice

Before CIA induction, we backcrossed *Sh3bp2^KI/+^* mice (C57BL/6 background) to the DBA/1 background for 10 generations. To investigate if P416R SH3BP2 heterozygous mutation affects the development of arthritis, we immunized *Sh3bp2^+/+^* (*n* = 15) and *Sh3bp2^KI/+^* (*n* = 14) mice with chick CII in complete Freund’s adjuvant. The incidence of arthritis at day 42 was comparable between *Sh3bp2^+/+^* and *Sh3bp2^KI/+^* mice (73.3% vs. 71.4%, respectively) (Figure 2B), although *Sh3bp2^KI/+^* mice showed a tendency of earlier development compared with *Sh3bp2^+/+^* mice (*P* = 0.13 at day 28 and *P* = 0.14 at day 31). These findings suggest that P416R SH3BP2 mutation does not significantly affect the immune events involved in the induction of arthritis. The severity of arthritis was significantly higher in arthritic *Sh3bp2^KI/+^* mice compared with arthritic *Sh3bp2^+/+^* mice at day 35–42 (12.83±1.02 in *Sh3bp2^KI/+^* vs. 8.07±0.94 in *Sh3bp2^+/+^* at day 42) (Figure 2C). The number of affected paws at day 35–42 was also significantly greater in arthritic *Sh3bp2^KI/+^* mice compared with *Sh3bp2^+/+^* mice (3.50±0.20 in *Sh3bp2^KI/+^* vs. 2.64±0.27 in *Sh3bp2^+/+^* at day 42) (Figure 2D). These results suggest that the SH3BP2 gain-of-function mutation augments inflammation in the CIA model.

### Exacerbated focal and systemic bone loss in *Sh3bp2^KI/+^* mice

**Figure 3:**
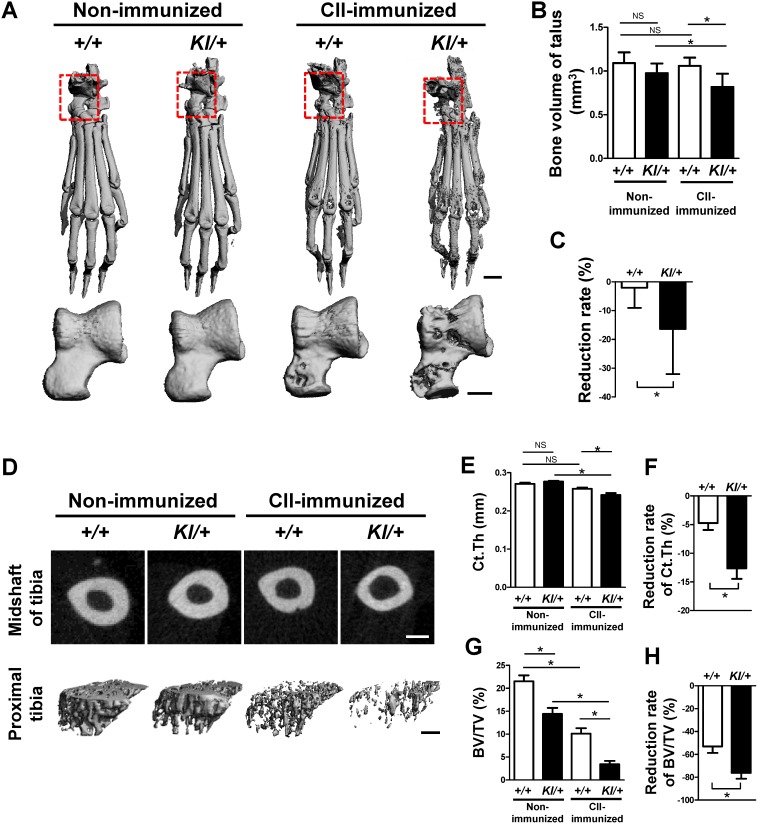
Increased focal and systemic bone loss in CII-immunized *Sh3bp2^KI/+^* mice. At 42 days after the initial immunization with CII, CII-immunized *Sh3bp2^+/+^* (*n* = 15) and *Sh3bp2^KI/+^* (*n* = 14) mice and age- and sex-matched non-immunized *Sh3bp2^+/+^* (*n* = 7) and *Sh3bp2^KI/+^* (*n* = 8) mice were euthanized. Focal and systemic bone loss was analyzed by micro-CT. **A**, Representative micro-CT images of hind paw and talus. **B**, Bone volume (BV) of talus. **C**, Reduction rate of BV of talus relative to non-immunized control mice of each genotype. **D**, Representative micro-CT images of cortical and trabecular bone of tibia. **E**, Cortical thickness (Ct.Th) of midshaft of tibia. **F**, Reduction rate of Ct.Th. **G**, Trabecular bone volume per total volume (BV/TV) of proximal tibia. **H**, Reduction rate of trabecular BV/TV. Scale bars: 1****mm for hind paw; 0.4****mm for talus and tibia. Values are presented as the mean ± SEM. *+/+* = *Sh3bp2^+/+^*; *KI/+* = *Sh3bp2^KI/+^*. * = *p*<0.05; NS = not significant.

Arthritic conditions cause focal bone loss in inflamed joints as well as systemic bone loss [42], [43]. To examine if gain of SH3BP2 function affects the focal and systemic bone loss, we analyzed the bone properties of the talus and the tibia as parameters for focal and systemic bone loss, respectively. CII-immunized *Sh3bp2^+/+^* (*n* = 15) and *Sh3bp2^KI/+^* (*n* = 14) male mice were euthanized at day 42. Age-matched non-immunized male *Sh3bp2^+/+^* (*n* = 7) and *Sh3bp2^KI/+^* (*n* = 8) mice were used as controls. We found that bone erosion on hind paws in CII-immunized *Sh3bp2^KI/+^* mice is augmented compared with *Sh3bp2^+/+^* mice (Figure 3A). The erosions were particularly evident around the metatarsophalangeal and ankle joints. The talar bone volume from CII-immunized *Sh3bp2^KI/+^* mice was smaller than that from CII-immunized *Sh3bp2^+/+^* mice (Figure 3B), and reduction rate of the bone volume relative to non-immunized controls was 9.5-fold greater in CII-immunized *Sh3bp2^KI/+^* mice compared with CII-immunized *Sh3bp2^+/+^* mice (Figure 3C).

We next examined the bone properties of the tibia to evaluate systemic bone loss. We found that the CII-immunized *Sh3bp2^KI/+^* mice show less cortical bone thickness (Ct.Th) at the midshaft and less trabecular bone volume per total volume (BV/TV) at the proximal tibia compared with CII-immunized *Sh3bp2^+/+^* mice (Figure 3D). Quantitative measurement of Ct.Th revealed that the reduction rate in CII-immunized mice relative to non-immunized controls is 2.7-fold greater in *Sh3bp2^KI/+^* mice (Figure 3E, 3F). Cortical BV/TV was also significantly decreased in CII-immunized *Sh3bp2^KI/+^* mice compared with *Sh3bp2^+/+^* mice (data not shown). Although non-immunized *Sh3bp2^KI/+^* mice showed a basal decrease in the trabecular BV/TV compared with non-immunized *Sh3bp2^+/+^* mice, BV/TV reduction rate of the CII-immunized mice relative to non-immunized controls was 1.4-fold greater in *Sh3bp2^KI/+^* mice (Figure 3G, 3H). These findings suggest that the gain of SH3BP2 function aggravates both focal and systemic bone loss in the CIA model.

### Enhanced inflammation, cartilage damage, and bone erosion in *Sh3bp2^KI/+^* mice

**Figure 4:**
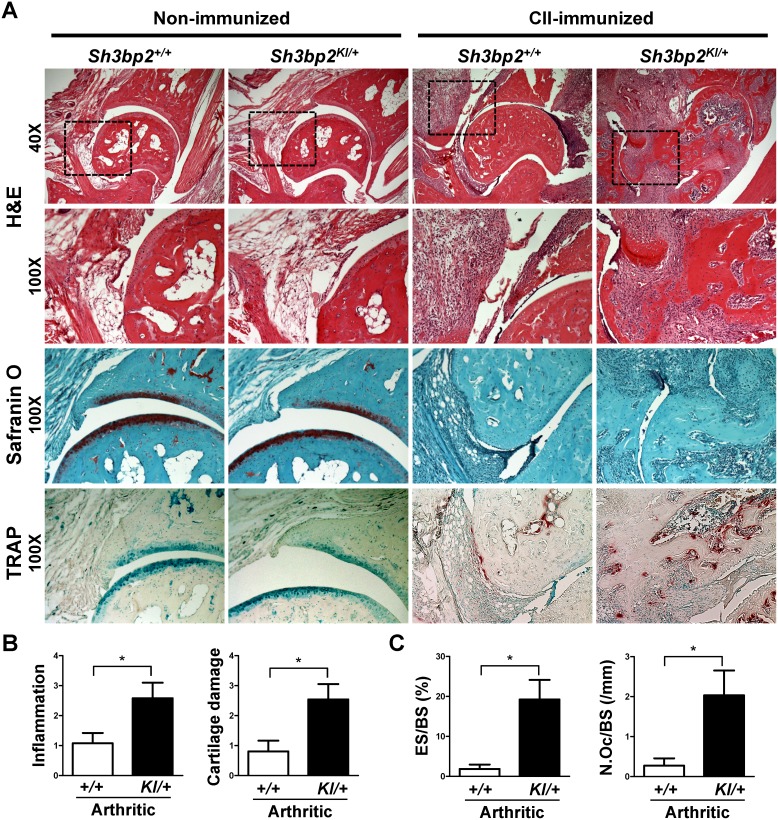
Increased inflammatory cell infiltration, cartilage damage, and bone erosion in tibiotalar joint of CII-immunized *Sh3bp2^KI/+^* mice. At 42 days after the initial immunization with CII, CII-immunized *Sh3bp2^+/+^* (*n* = 15) and *Sh3bp2^KI/+^* (*n* = 14) mice and age- and sex-matched non-immunized *Sh3bp2^+/+^* (*n* = 7) and *Sh3bp2^KI/+^* (*n* = 8) mice were euthanized. Inflammatory cell infiltration, cartilage damage, and bone erosion in tibiotalar joint were evaluated on hematoxylin and eosin (H&E), Safranin O, and tartrate-resistant acid phosphatase (TRAP) staining images, respectively. **A**, Representative serial H&E, Safranin O, and TRAP staining images of non-immunized and CII-immunized *Sh3bp2^+/+^* and *Sh3bp2^KI/+^* mice. Original magnifications: 40X (H&E, upper images) and 100X. **B**, Histological scores of inflammation and cartilage damage. Mean scores of arthritic *Sh3bp2^+/+^* (*n* = 11) and *Sh3bp2^KI/+^* (*n* = 10) mice. **C**, Histomorphometric analysis of talus. Eroded surface per bone surface (ES/BS) and number of osteoclasts per bone surface (N.Oc/BS) were determined. Values are presented as the mean ± SEM. *+/+* = *Sh3bp2^+/+^*; *KI/+* = *Sh3bp2^KI/+^*. * = *p*<0.05.

To analyze the inflamed joints histologically, we performed H&E, Safranin O, and TRAP staining of the ankle joints. Non-immunized *Sh3bp2^+/+^* and *Sh3bp2^KI/+^* mice did not exhibit inflammation and bone destruction in the joints (Figure 4A). Arthritic CII-immunized *Sh3bp2^KI/+^* mice developed more severe inflammatory cell infiltrates (H&E) and cartilage damage (Safranin O) and also exhibited increased osteoclast formation (TRAP) compared with those of arthritic *Sh3bp2^+/+^* mice (Figure 4A). Next, we quantified the severity of inflammation and cartilage damage using histological scoring systems. Both inflammation and cartilage damage in the joints were more severe in arthritic *Sh3bp2^KI/+^* mice (Figure 4B). To quantitatively evaluate osteoclast formation in the inflamed joints, we performed histomorphometric analysis of talar sections stained with TRAP. Most of the osteoclasts existed at the interface between pannus and erosion (Figure 4A, TRAP images). ES/BS and N.Oc/BS were increased in arthritic joints in *Sh3bp2^KI/+^* mice (Figure 4C), which is consistent with the greater reduction rate of talar bone volume in *Sh3bp2^KI/+^* mice (Figure 3B, 3C). These results suggest that increased bone erosion in inflamed joints of *Sh3bp2^KI/+^* mice is caused by enhanced osteoclast formation.

### Increased expression of TNF-α mRNA and osteoclast-associated genes in the inflamed joints of CII-immunized *Sh3bp2^KI/+^* mice

**Figure 5:**
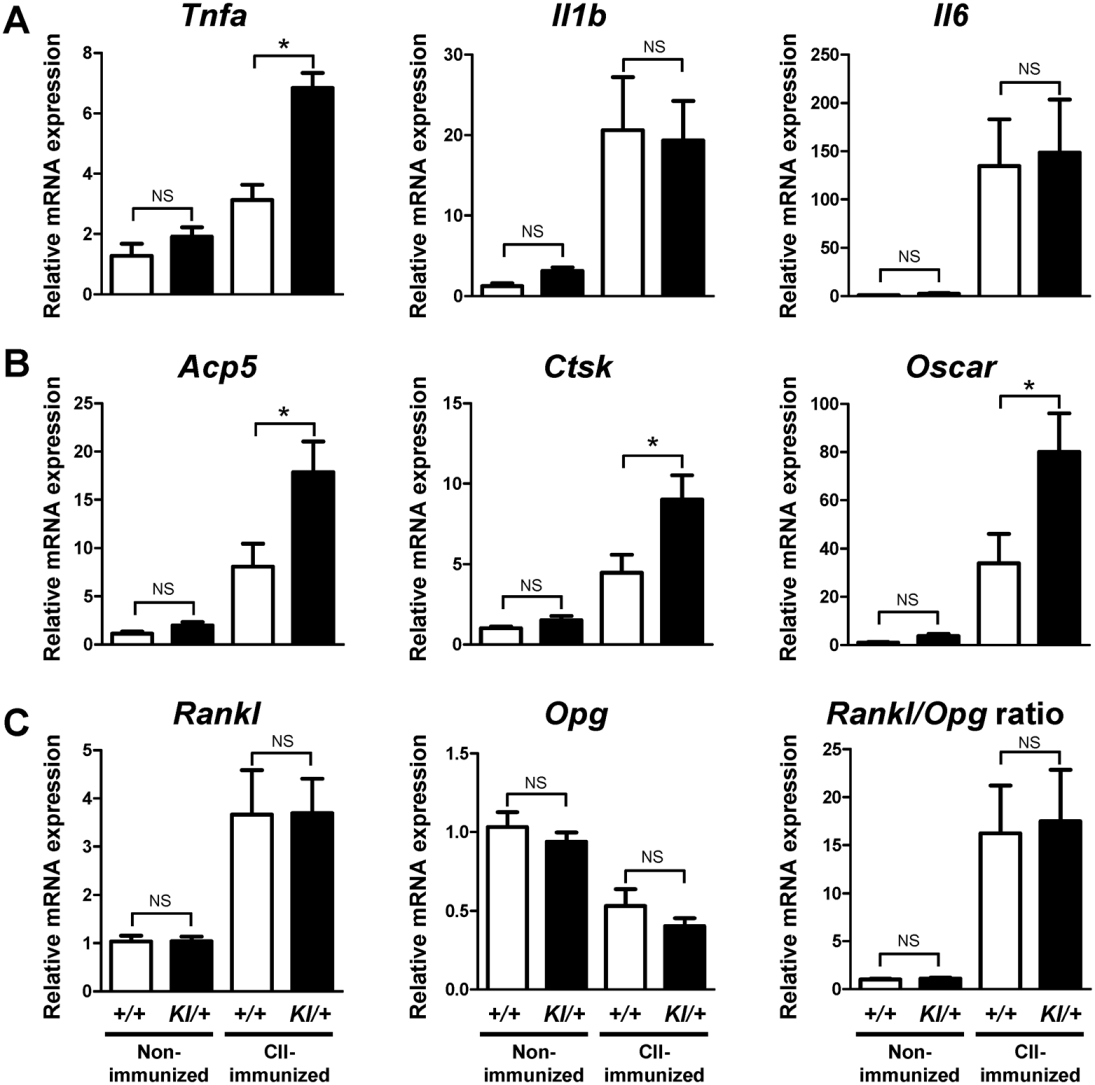
Increased gene expression of TNF-α and osteoclast-associated markers in ankle joint tissues of CII-immunized *Sh3bp2^KI/+^* mice. At 42 days after the initial immunization with CII, CII-immunized *Sh3bp2^+/+^* (*n* = 15) and *Sh3bp2^KI^* ^***/****+*^ (*n* = 14) mice and age- and sex-matched non-immunized *Sh3bp2^+/+^* (*n* = 7) and *Sh3bp2^KI/+^* (*n* = 8) mice were euthanized. RNA samples were isolated from the right ankle joint tissue and subjected to qPCR analysis. Gene expression levels of inflammatory cytokines (**A**), osteoclast-associated markers (**B**), and *Rankl*, *Opg*, and *Rankl/Opg* ratio (**C**) were determined. Gene expression levels relative to *Hprt* were calculated and normalized to the average expression level of non-immunized *Sh3bp2^+/+^* controls. Values are presented as the mean ± SEM. *+/+* = *Sh3bp2^+/+^*; *KI/+* = *Sh3bp2^KI/+^*. * = *p*<0.05; NS = not significant.

It is well known that proinflammatory cytokines such as TNF-α, IL-1β, and IL-6 play key roles in inflammatory bone destruction in RA. Therefore, we next examined the gene expression levels of inflammatory cytokines and osteoclast-associated markers in the ankle tissues at 42 days after CIA immunization. Arthritic condition increased the expression levels of *Tnfa*, *Il1b*, and *Il6* in both *Sh3bp2^+/+^* and *Sh3bp2^KI/+^* ankle tissues (Figure 5A). Among them, *Tnfa* level was significantly higher in CII-immunized *Sh3bp2^KI/+^* tissues than in CII-immunized *Sh3bp2^+/+^* tissues, while expression levels of *Il1b* and *Il6* were comparable between the CII-immunized *Sh3bp2^KI/+^* and *Sh3bp2^+/+^* tissues (Figure 5A). Expression levels of osteoclast-associated genes, acid phosphatase 5 (*Acp5*), cathepsin K (*Ctsk*), and osteoclast-associated receptor (*Oscar*), were significantly higher in ankle tissues from CII-immunized *Sh3bp2^KI/+^* mice compared with those from CII-immunized *Sh3bp2^+/+^* mice (Figure 5B). Expression levels of *Rankl* and osteoprotegerin (*Opg*), and *Rankl/Opg* ratio were similar in ankle tissues from CII-immunized *Sh3bp2^KI/+^* and *Sh3bp2^+/+^* mice (Figure 5C). These findings suggest that CII-immunized *Sh3bp2^KI/+^* mice develop greater TNF-α-driven inflammation associated with increased osteoclastogenesis, at least, at day 42 after the immunization.

### No significant abnormality in proliferation and in IFN-γ and IL-17 production in *Sh3bp2^KI/+^* draining lymph node cell culture

**Figure 6:**
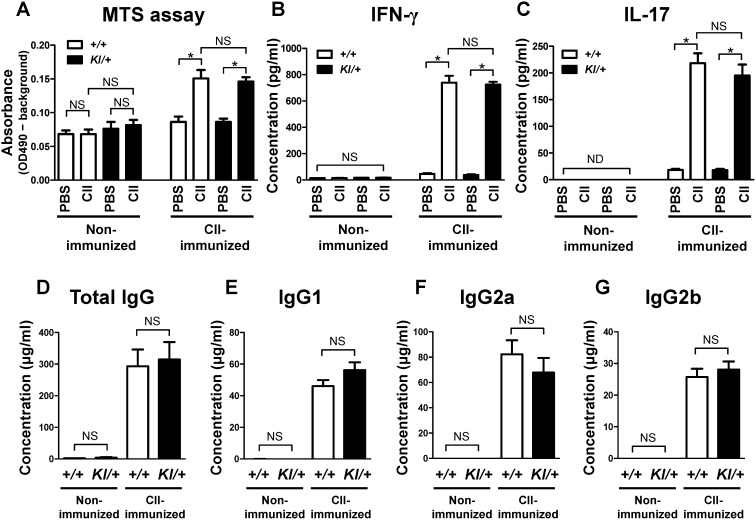
Proliferation and cytokine production in lymph node cell culture and serum levels of anti-mouse CII antibody. **A**–**C**, Inguinal lymph nodes were isolated at 10 days after immunization with chick CII. Lymph node cells (4×10^5^ cells/well) were stimulated with chick CII (50 µg/ml) for 72 hours. Proliferation of the cells was determined by a colorimetric assay using MTS reagent (**A**). Levels of IFN-γ (**B**) and IL-17 (**C**) in culture supernatant were measured by ELISA. The lower limit of detection is 10–20 pg/ml. **D–G**, Serum samples were collected at 42 days after initial immunization, and serum samples from age- and sex-matched mice were collected as controls. Total IgG, IgG1, IgG2a, and IgG2b against mouse CII were measured by ELISA (*n* = 7–15/group). Values are presented as the mean ± SEM. *+/+* = *Sh3bp2^+/+^*; *KI/+* = *Sh3bp2^KI/+^*. * = *p*<0.05; NS = not significant; ND = not detectable.

T cells play critical roles in the initiation of arthritis in the CIA model, and T cell-derived cytokines such as IFN-γ and IL-17 control the activation of macrophages and osteoclasts directly or indirectly [44]–[46]. To evaluate if the gain of SH3BP2 function regulates T cell function, inguinal lymph node cells were isolated from CII-immunized mice. Proliferation and IFN-γ and IL-17 production by the cells in response to CII were determined. We found that cell proliferation after CII stimulation is comparable between CII-immunized *Sh3bp2^+/+^* and *Sh3bp2^KI/+^* cells (Figure 6A) and that IFN-γ and IL-17 levels in the culture medium are also similar in CII-immunized *Sh3bp2^+/+^* and *Sh3bp2^KI/+^* cells (Figure 6B, 6C), suggesting that the gain of SH3BP2 function does not significantly affect the responsiveness of T cells to CII.

### No significant abnormality in serum anti-mouse CII antibody levels in *Sh3bp2^KI/+^* mice

Autoantibody production by B cells is important for the development of arthritis in the CIA model [44], [47]. To examine if the gain of SH3BP2 function affects the autoantibody production, we determined the levels of anti-mouse CII antibody in serum at 42 days after initial immunization. Serum anti-CII antibody (total IgG) levels were comparable between CII-immunized *Sh3bp2^+/+^* and *Sh3bp2^KI/+^* mice (Figure 6D). We also found that the levels of IgG subclasses, IgG1, IgG2a, and IgG2b, are similar in CII-immunized *Sh3bp2^+/+^* and *Sh3bp2^KI/+^* mice (Figure 6E–G). These findings suggest that the gain of SH3BP2 function does not significantly influence the autoantibody production in the CIA model.

### Increased TNF-α production in *Sh3bp2^KI/+^* macrophages

**Figure 7:**
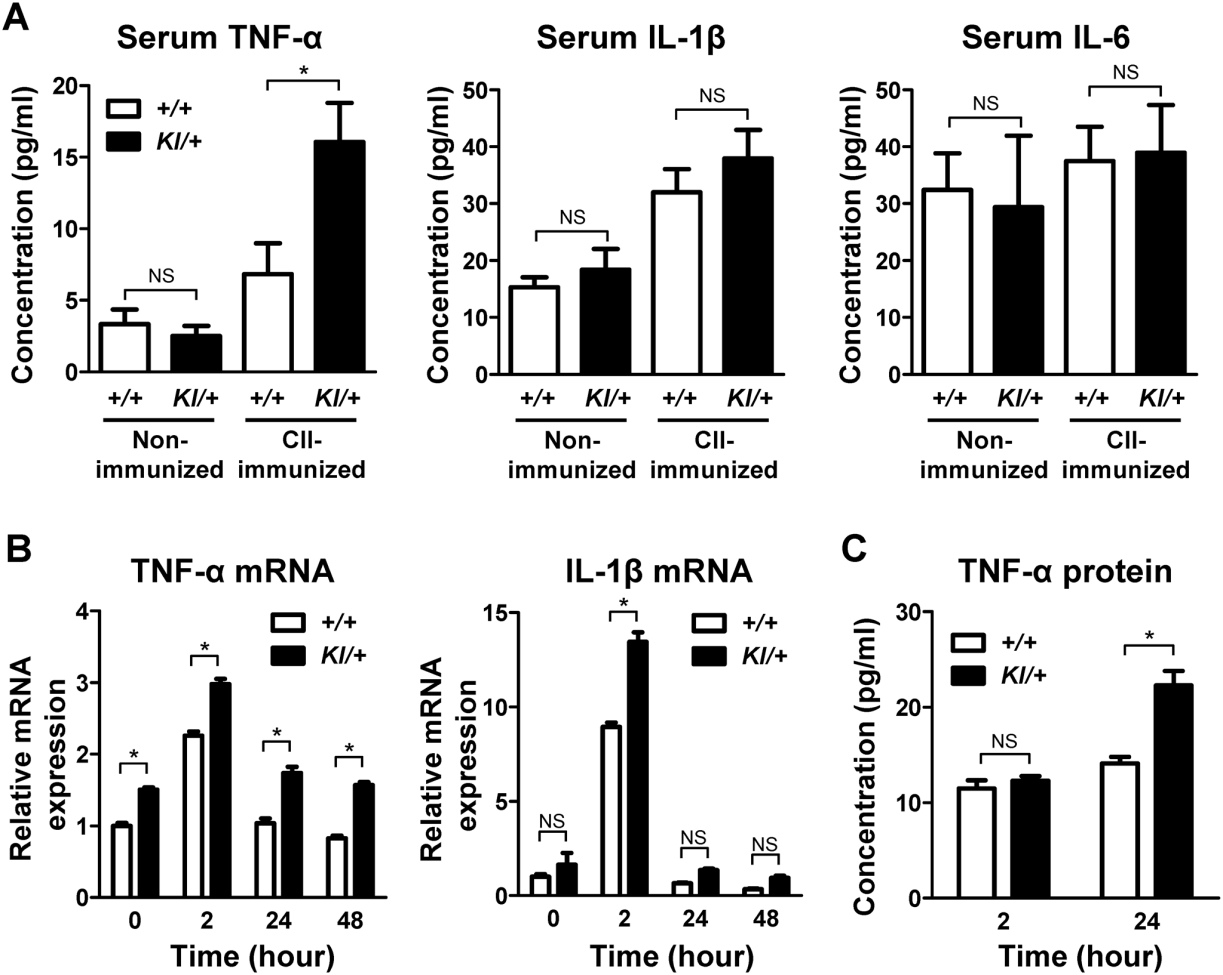
Increased serum TNF-α in *Sh3bp2^KI/+^* mice and increased TNF-α expression in *Sh3bp2^KI/+^* bone marrow-derived macrophage culture. **A**, Serum TNF-α, IL-1β, and IL-6 levels in CII-immunized mice. Serum samples were collected at 42 days after CII immunization from CII-immunized *Sh3bp2^+/+^* (*n* = 15) and *Sh3bp2^KI^* ^***/****+*^ (*n* = 14) mice and from age- and sex-matched non-immunized *Sh3bp2^+/+^* (*n* = 7) and *Sh3bp2^KI/+^* (*n* = 8) mice. Serum TNF-α, IL-1β, and IL-6 levels were determined by ELISA. **B–C**, Primary bone marrow cells were isolated from long bones of *Sh3bp2^+/+^* and *Sh3bp2^KI/+^* mice. Non-adherent bone marrow cells were seeded at a density of 1.0**×**10^5^/well on 48-well plates. After 2-day preculture with M-CSF (25****ng/ml), BMMs were re-stimulated with M-CSF (25****ng/ml). TNF-α and IL-1β mRNA expression levels in BMMs were quantified at the indicated time points by qPCR analysis (**B**). Gene expression levels relative to *Hprt* were calculated and normalized to the expression levels of *Sh3bp2^+/+^* baseline control at 0 hour. TNF-α protein levels in culture supernatants were determined by ELISA (C). Values are presented as the mean ± SEM. *+/+* = *Sh3bp2^+/+^*; *KI/+* = *Sh3bp2^KI/+^*. * = *p*<0.05; NS = not significant.

TNF-α plays a major role in the pathogenesis of RA [6]–[8]. As shown in Figure 5A, TNF-α expression in the inflamed *Sh3bp2^KI/+^* joints is elevated compared with that in the inflamed *Sh3bp2^+/+^* joints. Then we measured serum TNF-α levels in the CII-immunized mice. We found that serum TNF-α levels are higher in CII-immunized *Sh3bp2^KI/+^* mice than in CII-immunized *Sh3bp2^+/+^* mice (Figure 7A). In contrast, serum IL-1β and IL-6 levels were comparable between CII-immunized *Sh3bp2^KI/+^* and *Sh3bp2^+/+^* mice (Figure 7A).

Macrophages are the major source of TNF-α in inflammatory bone diseases [48]. Since M-CSF contributes to the development of CIA [49] and its expression is upregulated in fibroblast-like synoviocytes, T cells, and endothelial cells in RA [50], [51], we examined if TNF-α expression is increased in the *Sh3bp2^KI/+^* macrophages in response to M-CSF. TNF-α mRNA expression levels were higher in *Sh3bp2^KI/+^* BMMs compared with *Sh3bp2^+/+^* BMMs throughout the culture period (Figure 7B). Furthermore, TNF-α protein levels in the culture supernatant of *Sh3bp2^KI/+^* BMMs were increased at 24 hours after M-CSF stimulation (Figure 7C). In contrast, IL-1β mRNA levels were comparable in *Sh3bp2^KI/+^* and *Sh3bp2^+/+^* BMMs except at 2 hours after M-CSF stimulation (Figure 7B) and those of IL-6 were below the detection limit in *Sh3bp2^KI/+^* and *Sh3bp2^+/+^* BMMs (data not shown). Collectively, these results suggest that increased TNF-α levels in the joints and serum of the CIA *Sh3bp2^KI/+^* mice are, at least in part, due to the elevated activation of *Sh3bp2^KI/+^* macrophages compared with *Sh3bp2^+/+^* macrophages. These results are consistent with our previous report, which showed that *Sh3bp2^KI/+^* BMMs in C57BL/6 genetic background produce a greater amount of TNF-α in response to M-CSF [23].

### Enhanced RANKL-induced osteoclastogenesis in *Sh3bp2^KI/+^* macrophages via increased NFATc1 nuclear translocation

**Figure 8:**
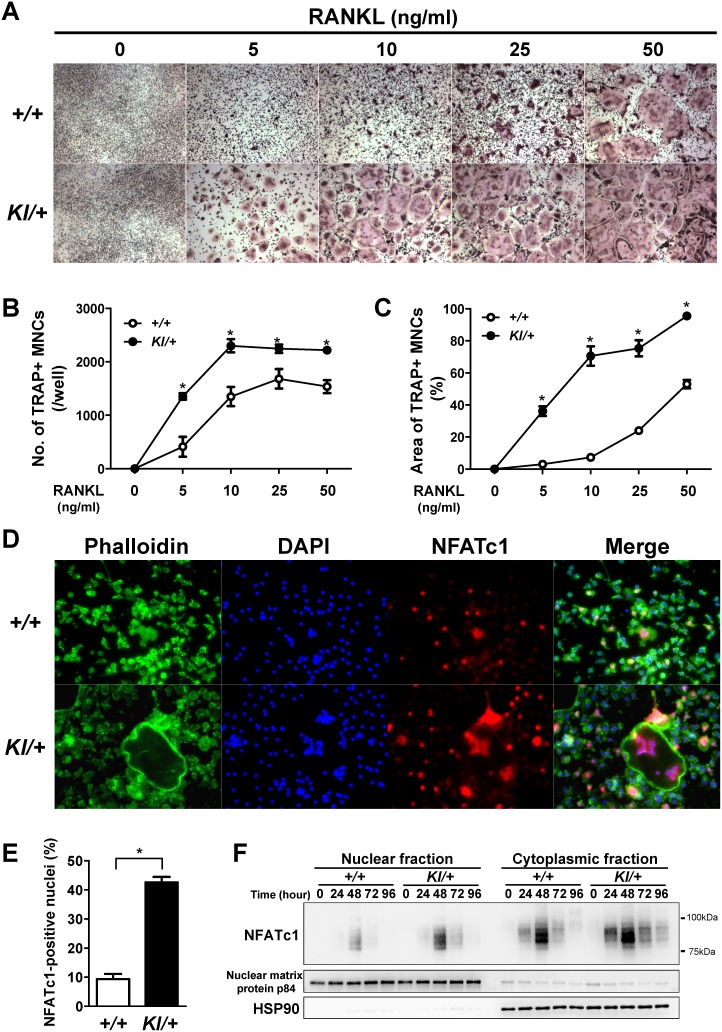
Increased RANKL-induced osteoclastogenesis in *Sh3bp2^KI/+^* bone marrow-derived macrophage culture. Primary bone marrow cells were isolated from long bones of *Sh3bp2^+/+^* and *Sh3bp2^KI/+^* mice. Non-adherent bone marrow cells were seeded at a density of 1.0**×**10^5^/well on 48-well plates. **A–C**, After 2-day preculture with M-CSF (25****ng/ml), BMMs were stimulated with RANKL at indicated concentrations for 3 days. Formation of TRAP-positive multinucleated cells (TRAP+ MNCs) was evaluated by TRAP staining (**A**). Original magnification: 40X. Number of TRAP+ MNCs per well (**B**) and area of TRAP+ MNCs per well (**C**) were determined. **D–E**, Immunofluorescent staining of actin, nuclei, and NFATc1. After 2-day preculture with M-CSF (25****ng/ml), BMMs were stimulated with RANKL (25****ng/ml) in the presence of M-CSF for 48 hours. Actin, nuclei, and NFATc1 were visualized by phalloidin, DAPI, and anti-NFATc1 antibody, respectively (**D**). Original magnification: 200X. The percentage of NFATc1-positive nuclei per total nuclei was determined (**E**). **F**, Western blot analysis for NFATc1. After 2-day preculture with M-CSF (25****ng/ml), BMMs were stimulated with RANKL (10****ng/ml) in the presence of M-CSF for the indicated periods. NFATc1 expression levels in nuclear and cytoplasmic fractions were determined. Nuclear matrix protein p84 and heat shock protein 90 (HSP90) were used as loading controls. Values are presented as the mean ± SEM. *+/+* = *Sh3bp2^+/+^*; *KI/+* = *Sh3bp2^KI/+^*. * = *p*<0.05.

Consistent with previous reports [23], [37], *Sh3bp2^KI/+^* BMMs formed more TRAP+ MNCs in response to RANKL than *Sh3bp2^+/+^* BMM did (Figure 8A). Increased numbers and areas of TRAP+ MNCs in *Sh3bp2^KI/+^* BMM cultures were confirmed by quantitative analyses (Figure 8B, 8C). These results suggest that *Sh3bp2^KI/+^* BMMs on DBA/1 background is susceptible to RANKL stimulation. Lastly, we examined the mechanism by which gain of SH3BP2 function increases TRAP+ MNCs formation. Osteoclast differentiation is regulated by various transcription factors including NFATc1, MITF, c-Fos, and NF-κB [52]. Among them, NFATc1 is known as a master regulator of osteoclastogenesis [53]. Since NFATc1 functions downstream of SH3BP2 [37], we evaluated the nuclear localization of NFATc1 in RANKL-stimulated BMMs. BMMs were stimulated with RANKL (25 ng/ml) for 48 hours, and NFATc1 was visualized by immunofluorescent staining. As shown in Figure 8D, NFATc1 was highly expressed predominantly in the nuclei of RANKL-stimulated *Sh3bp2^+/+^* and *Sh3bp2^KI/+^* BMMs. The percentage of NFATc1-positive nuclei was significantly greater in *Sh3bp2^KI/+^* BMMs than in *Sh3bp2^+/+^* BMMs (Figure 8E). Western blot analysis also revealed an increased NFATc1 nuclear localization in *Sh3bp2^KI/+^* BMMs compared with *Sh3bp2^+/+^* BMMs (Figure 8F). Taken together, these results indicate that the gain of SH3BP2 function accelerates RANKL-induced osteoclastogenesis in BMMs via enhanced nuclear localization of NFATc1.

## Discussion

We undertook this study to test our hypothesis that SH3BP2 plays a role in the development of immune-mediated systemic arthritis. For this purpose, CIA was induced in heterozygous P416R SH3BP2 mutant mice, in which SH3BP2 functions in a gain-of-function manner through an increased level of SH3BP2 protein. We demonstrated that the P416R SH3BP2 mutation enhances the severity of inflammation and augments focal and systemic bone loss. These findings highlight the critical participation of SH3BP2 in synovial inflammation and bone loss in this animal model.

SH3BP2 is expressed in various immune cells such as T cells, B cells, and macrophages as well as osteoclasts, indicating that SH3BP2 potentially regulates both immune and skeletal systems [13]–[17], [23]. Since inflammatory bone loss is mediated by complex interactions involving immune and bone cells [6]–[8], we investigated how SH3BP2 regulates the development of joint inflammation and bone destruction. Our data showed that T cell activation and autoantibody production in response to CII immunization are not noticeably altered by P416R SH3BP2 mutation (Figure 6A–G), suggesting that the SH3BP2 gain-of-function mutation is likely to exacerbate the severity of inflammation and bone loss independently of T and B cells. Importantly, our study demonstrated that the number of osteoclasts as well as the expression of TNF-α and osteoclast-associated genes are increased in the inflamed joints from *Sh3bp2^KI/+^* mice (Figure 4A, 4C, 5A, and 5B). We also showed that serum TNF-α is increased in the CII-immunized *Sh3bp2^KI/+^* mice (Figure 7A) and that TNF-α production is increased in *Sh3bp2^KI/+^* BMMs (Figure 7B, 7C). Moreover, we revealed that *Sh3bp2^KI/+^* BMMs form more osteoclasts through increased NFATc1 nuclear translocation (Figure 8). Taken together, these results suggest that gain of SH3BP2 function plays roles, at least, in macrophages and osteoclasts, leading to enhanced inflammatory bone loss in the CIA model.

Our study shows that osteoclast differentiation is enhanced in the inflamed joints of *Sh3bp2^KI/+^* mice compared with *Sh3bp2^+/+^* mice (Figure 4A, 4C, and 5B), although *Rankl* expression levels are comparable between the *Sh3bp2^+/+^* and *Sh3bp2^KI/+^* joints (Figure 5C). The increased osteoclast differentiation in *Sh3bp2^KI/+^* mice can be explained by three different mechanisms. First, the P416R SH3BP2 mutation enhances the responsiveness of osteoclast precursors to RANKL (Figure 8). Second, the P416R SH3BP2 mutation enhances TNF-α mRNA expression in inflamed joints compared with that of *Sh3bp2^+/+^* mice (Figure 5A). This increased TNF-α expression in *Sh3bp2^KI/+^* joints may augment osteoclast formation synergistically with RANKL [9]. Third, we have recently discovered that the P416R SH3BP2 mutation enhances TNF-α-induced osteoclastogenesis independently of RANKL [34]. This increased susceptibility of osteoclast precursors to TNF-α could also be involved in the enhanced osteoclastogenesis in the CII-immunized *Sh3bp2^KI/+^* mice. Therefore, we propose that a cumulative effect of these mechanisms is responsible for the increased osteoclast formation and subsequent bone loss in CII-immunized *Sh3bp2^KI/+^* mice. However, it should be noted that SH3BP2 is known to be involved in the diverse cell functions in various types of cells [13], [17], [19], [20], [54], [55], thereby we cannot exclude the possibility that other immune cells (e.g. NK cells, neutrophils, mast cells) as well as stromal cells (e.g. osteoblasts, synovial fibroblasts) are also involved in the exacerbated bone loss in *Sh3bp2^KI/+^*mice in the CIA model.

It has been reported that SH3BP2 plays roles in T-cell [18], [56] and B-cell responses [14], [15], [57]–[59]. However, SH3BP2 gain-of-function mutation did not alter the T- and B-cell responses in the CIA model (Figure 6). This may be explained by the following reasons; (1) Roles of SH3BP2 varied depending on the *in vitro* and *in vivo* models. (2) Differences in genetic background affected the necessity of the SH3BP2 in T- and B-cell functions. The C57BL/6 mice were used in the previous studies [14], [15], while DBA/1 mice were used in our current study. (3) SH3BP2 gain-of-function also activated negative feedback pathways that suppress T- and B-cell responses. (4) The P416R SH3BP2 mutation played a role in the activation of T and B cells, but autoantibody and cytokine productions were saturated due to very high susceptibility to CIA of DBA/1 mice. Therefore, we were not able to detect the increased activation by measuring the autoantibody and cytokine levels. Further analyses will be required to determine whether SH3BP2 gain-of-function augments T- and B-cell functions and whether it has a role in the exacerbation of inflammatory bone loss via T and B cells in the CIA model.

Our data suggest that increased TNF-α level in serum and joint of CII-immunized *Sh3bp2^KI/+^* mice (Figure 5A, 7A) is a potential cause of the exacerbated bone loss by osteoclasts. However, we are not able to exclude the possibility that pro-inflammatory cytokines other than TNF-α contribute to the enhanced osteoclast formation in CII-immunized *Sh3bp2^KI/+^* mice. Indeed, IL-1β mRNA expression was elevated in *Sh3bp2^KI/+^* BMMs at 2 hours after M-CSF stimulation (Figure 7B), although a significant increase in IL-1β levels in serum and joint was not observed at day 42 in CII-immunized *Sh3bp2^KI/+^* mice. Dynamic expression pattern of inflammatory cytokines during the CIA development is reported [36]. Therefore, IL-1β expression might be higher at different time points in CII-immunized *Sh3bp2^KI/+^* mice, which might exacerbate the CIA development in the *Sh3bp2^KI/+^* mice.

Our data also show that mutant SH3BP2 plays a key role in TNF-α production in macrophages. Interestingly, macrophages are highly heterogeneous cells that can rapidly change their function in response to signals in microenvironment [60]. Therefore, it may be possible that mutant SH3BP2 regulates macrophage functions other than TNF-α production such as antigen-presentation in CIA development. Further analysis of CIA in *Sh3bp2^KI/+^* mice focusing on the diverse functions of macrophages would lead to the discovery of the novel function of SH3BP2 in autoimmune arthritis.

In humans, development of cherubism lesions is limited to the lower face. Involvement of extracranial bones has not been reported except a few cases with isolated lesions in humerus and ribs [25], [61]–[63]. To date there is no report on cherubism patients who developed arthritis. This might be due to the lack of long-term follow-up, because cherubism is a rare disease and cherubism lesions spontaneously regress after puberty. Our study demonstrates that the P416R heterozygous mutation augments inflammation and bone loss in mice immunized with the arthritogenic antigen. If this is the case in humans, cherubism patients may exhibit more severe inflammation and bone loss if they develop immune-mediated arthritis.

Heterozygous SH3BP2 mutations are responsible for cherubism in human [24]. On the other hand, homozygous mutation is required to develop cherubism-like jawbone destruction in the mouse model of cherubism [23], [64]. In this study, CII-immunized *Sh3bp2^KI/+^* mice developed more severe joint destruction than *Sh3bp2^+/+^* mice. However, neither CII-immunized *Sh3bp2^KI/+^* nor *Sh3bp2^+/+^* mice developed jawbone destruction (data not shown). This indicates that jaw-targeted stimulation such as with oral bacterial pathogens might be required for *Sh3bp2^KI/+^* mice to develop cherubism-like jawbone destruction.

This unique genetically mutated mouse model may provide valuable insights into the pathogenesis of RA. It is clinically well recognized that the severity of radiologic progression is variable between RA patients. Several genetic factors have been identified, which partly explain the variance in radiologic progression [65]–[67]. Although a direct association between SH3BP2 and RA has not yet been identified [68], genetic variations that affect the expression level of SH3BP2 may be involved in the severity of bone loss progression in RA, because loss- and gain-of-function of SH3BP2 oppositely regulate RANKL-induced osteoclastogenesis [20], [23]. The present study demonstrates that a single gene mutation affects the development of inflammatory bone loss in the immune-mediated systemic arthritis. Further analysis is needed to determine whether activation of SH3BP2-mediated pathways in macrophages and osteoclasts regulates the structural changes of RA and other forms of inflammatory bone diseases.

In conclusion, the P416R SH3BP2 gain-of-function mutation exacerbates inflammation as well as focal and systemic bone loss in the murine CIA model by increasing TNF-α production in macrophages and RANKL-induced osteoclastogenesis. These findings suggest that SH3BP2 plays a role in the pathogenesis of arthritis and that, in contrast to the gain-of-function effect of SH3BP2, suppression of SH3BP2 function may effectively reduce inflammation and bone loss in inflammatory bone destructive diseases.
